# Supplementary material for: An adenovirus-vectored COVID-19 vaccine confers protection from SARS-COV-2 challenge in rhesus macaques
Source: Nat Commun. 2020 Aug 21;11:4207. doi: 10.1038/s41467-020-18077-5 (PMC7442803; doi:10.1038/s41467-020-18077-5)
Supplement: Supplementary file 1 — Supplementary Information [file 41467_2020_18077_MOESM1_ESM.pdf]

Supplementary Information File for:

An adenovirus-vectored COVID-19 vaccine confers protection from SARS-COV-2 challenge in rhesus macaques

Feng L et al.

Supplementary Figures and the legends

Supplementary Figure 1

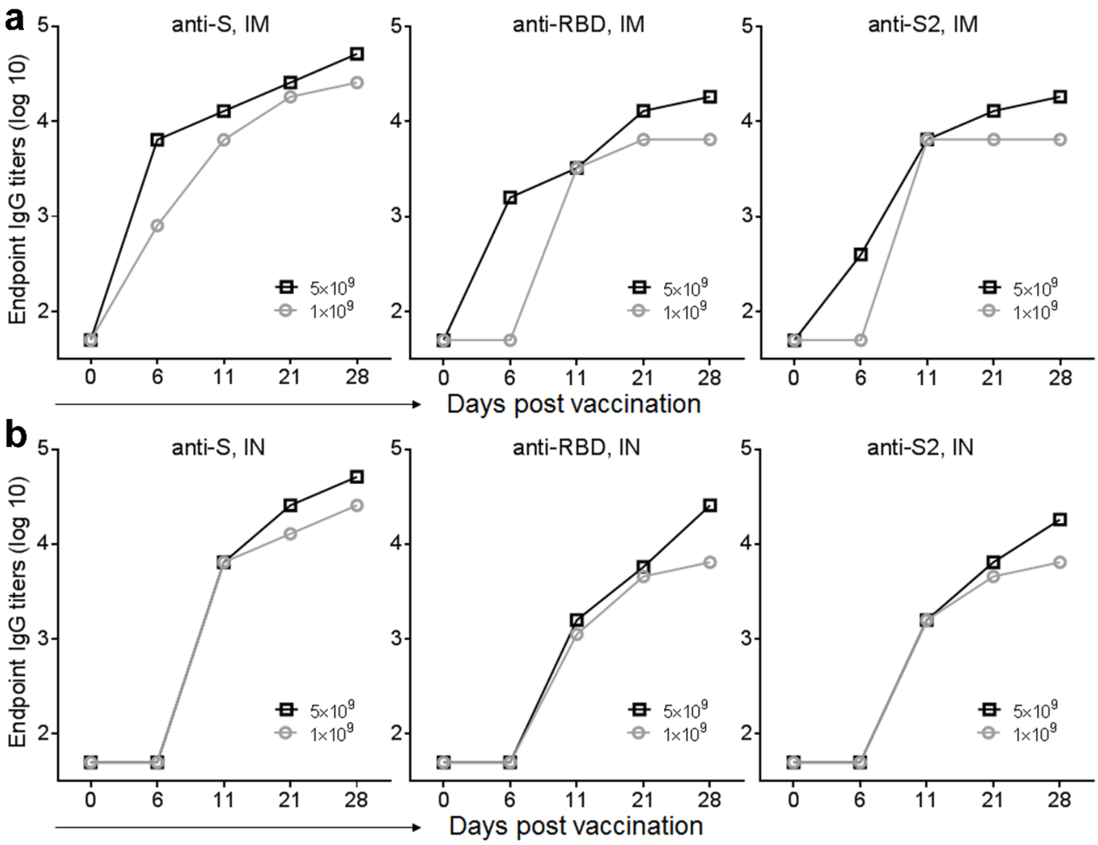

Supplementary Figure 1. Dynamics of S-binding IgG antibodies in Ad5-S-nb2 vaccinated mice. a IgG antibodies against the S protein, RBD and the S2 subunit in mice IM vaccinated with Ad5-S-nb2 on days 0, 6, 11, 21, and 28 after vaccination. **b** IgG antibodies against the S protein, RBD and the S2 subunit in mice IN vaccinated with Ad5-S-nb2 on days 0, 6, 11, 21, and 28 after vaccination. The serum samples from each mouse in one group (n = 5) were equally pooled and examined by ELISA. The data points indicate the mean value of two technical replicates. Source data are provided as a Source Data file.

Supplementary Figure 2

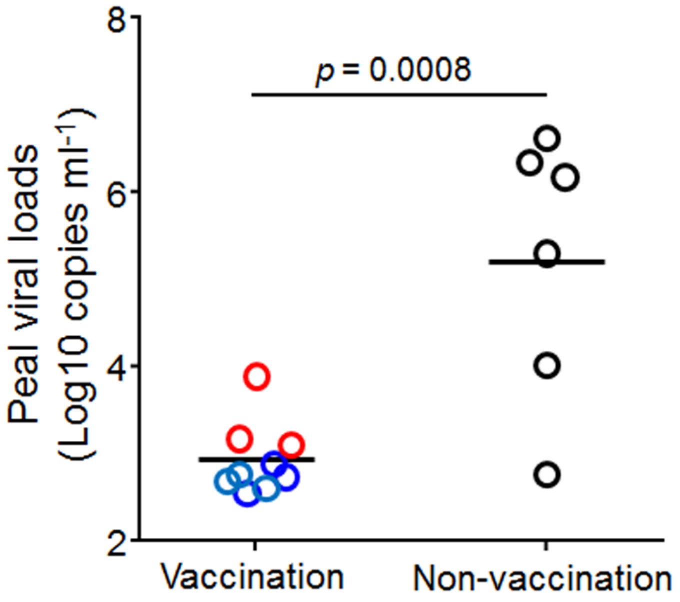

Supplementary Figure 2. Peak viral loads in the pharyngeal swabs of vaccinated macaques and non-vaccinated macaques. Each circle represents the peak viral load of one macaque. Blue circle, macaques that received IM vaccination with 1×10^{11} vp Ad5-S-nb2; Red circles, macaques that received IN vaccination with 5×10^{10} vp Ad5-S-nb2; Dark cyan circles, macaques that received IM vaccination with 1×10^{10} vp Ad5-S-nb2; Black circles, non-vaccinated macaques. Black lines reflect the mean viral loads. Comparison between vaccinated (n = 9) and non-vaccinated (n = 6) macaques was conducted using Student's t-test (unpaired, two-tailed). Source data are provided as a Source Data file.

Supplementary Figure 3

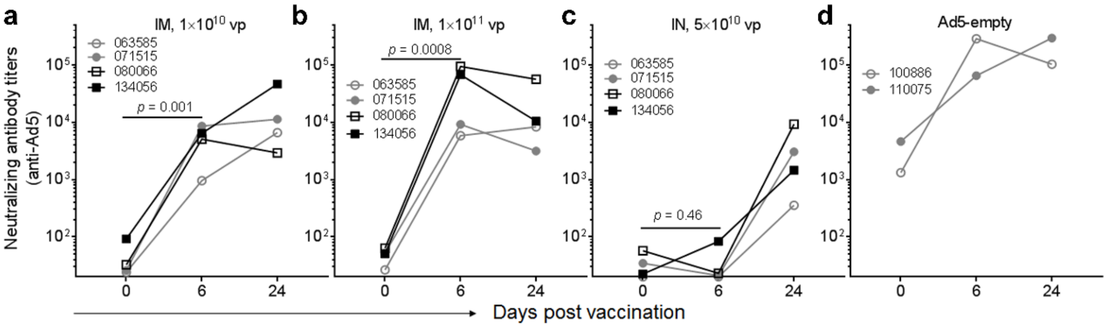

Supplementary Figure 3. Neutralizing antibodies against Ad5 in macaques before and after vaccination. a Dynamics of anti-Ad5 neutralizing antibodies in macaques that received IM vaccination with 1×10^{11} vp Ad5-S-nb2. **b** Dynamics of anti-Ad5 neutralizing antibodies in macaques that received IM vaccination with 1×10^{10} vp Ad5-S-nb2. **c** Dynamics of anti-Ad5 neutralizing antibodies in macaques that received IN vaccination with 5×10^{10} vp Ad5-S-nb2. **d** Dynamics of anti-Ad5 neutralizing antibodies in macaques that received IM vaccination with 1×10^{11} vp Ad5-empty. Macaque serum samples were assessed using a neutralization assay based on an Ad5-SEAP reporter virus. Each data point represents the mean value of two technical replicates. Comparison between different time points were conducted using Student's t-test (paired, two-tails, n = 4). Source data are provided as a Source Data file.

Supplementary Table 1.

Macaque ID	Vaccination		Body weight (kg)	Age (year)	Sex	Immunisation history						
	Dose	Route				2013	2014	2015	2016	2017	2018	2019
063585	Ad5-S-nb2 1×10^{10} vp	IM	12.2	14	male	IIV(H7N7,H7N9)[a]	IIV(H7N7,H7N9)	IIV(H7N9)	—	IIV(H5N6)	IIV(H5N6)	—
071515			12.1	13	male	IIV(H7N9)	IIV(H7N9)	—	—	IIV(H5N6)	IIV(H5N6)	—
080066			8.5	12	female	IIV(H7N9)	IIV(H7N9)	—	—	IIV(H5N6)	IIV(H5N6)	
134056			6.2	7	female	—	—	—	—	—	—	IIV(H1N1,H7N9), Ad2-EBOLA GP[b]
100109	Ad5-S-nb2 1×10^{11} vp	IM	9.7	10	male	—	—	—	—	IIV(H5N6)	IIV(H5N6)	—
116004			16.7	9	male	—	—	—	—	IIV(H5N6)	IIV(H5N6)	—
116008			6.6	9	female	—	—	—	—	IIV(H5N6)	IIV(H5N6)	—
130460			5.7	7	female	—	—	—	—	—	—	IIV(H1N1,H7N9), Ad2-EBOLA GP
071539	Ad5-S-nb2 5×10^{10} vp	IN	10.2	13	male	IIV(H7N9)	IIV(H7N9)	—	—	IIV(H5N6)	IIV(H5N6)	—
110113			9.3	9	male	—	—	—	—	IIV(H5N6)	IIV(H5N6)	—
140052			6.2	6	female	—	—	—	—	—	—	IIV(H1N1,H7N9), Ad2-EBOLA GP
134018			7.1	7	female	—	—	—	—	—	—	IIV(H1N1,H7N9), Ad2-EBOLA GP
100886	Ad5-empty 1×10^{11} vp	IM	7.3	10	female	—	EBOLA VLP[c]	EBOLA VLP, Ad2-EBOLA GP	EBOLA GP[d], Ad2-EBOLA GP	EBOLA GP	EBOLA GP, IIV(H7N9)	Ad5-empty
110075			6.8	9	male	—	EBOLA VLP	EBOLA VLP	EBOLA GP, EBOLA VLP	EBOLA GP, Ad2-EBOLA GP	EBOLA GP, IIV(H7N9)	Ad5-empty
C1	Non-vaccinated	——	6.7	7	female	——						
C2			9.3	9	male	——						
C3			6.3	6	female	——						
C4			9.0	7	male	——						
D1			6.6	7	female	——						
D2			10.1	9	male	——						

[a]IIV, Inactivated influenza virus.

[b]An adenovirus type 2 vector expressing the glycoprotein (GP) of Ebola virus.

[c]Virus like particles harboring the GP protein of Ebola virus.

[d]Purified GP protein of Ebola virus.
