# Supplementary material for: An adenovirus-vectored COVID-19 vaccine confers protection from SARS-COV-2 challenge in rhesus macaques
Source: Nat Commun. 2020 Aug 21;11:4207. doi: 10.1038/s41467-020-18077-5 (PMC7442803; doi:10.1038/s41467-020-18077-5)
Supplement: Supplementary file 3 — Reporting Summary [file 41467_2020_18077_MOESM3_ESM.pdf]

nature research

Corresponding author(s): Suhua Guan, Zhiming Yuan, Ling Chen

Last updated by author(s): Jul 23, 2020

Reporting Summary

Nature Research wishes to improve the reproducibility of the work that we publish. This form provides structure for consistency and transparency in reporting. For further information on Nature Research policies, see our Editorial Policies and the Editorial Policy Checklist.

Statistics

For all statistical analyses, confirm that the following items are present in the figure legend, table legend, main text, or Methods section.

n/a	Confirmed	
☐	☒	The exact sample size (*n*) for each experimental group/condition, given as a discrete number and unit of measurement
☐	☒	A statement on whether measurements were taken from distinct samples or whether the same sample was measured repeatedly
☐	☒	The statistical test(s) used AND whether they are one- or two-sided<br>*Only common tests should be described solely by name; describe more complex techniques in the Methods section.*
☒	☐	A description of all covariates tested
☒	☐	A description of any assumptions or corrections, such as tests of normality and adjustment for multiple comparisons
☐	☒	A full description of the statistical parameters including central tendency (e.g. means) or other basic estimates (e.g. regression coefficient) AND variation (e.g. standard deviation) or associated estimates of uncertainty (e.g. confidence intervals)
☐	☒	For null hypothesis testing, the test statistic (e.g. *F*, *t*, *r*) with confidence intervals, effect sizes, degrees of freedom and *P* value noted<br>*Give P values as exact values whenever suitable.*
☒	☐	For Bayesian analysis, information on the choice of priors and Markov chain Monte Carlo settings
☒	☐	For hierarchical and complex designs, identification of the appropriate level for tests and full reporting of outcomes
☒	☐	Estimates of effect sizes (e.g. Cohen's *d*, Pearson's *r*), indicating how they were calculated

Our web collection on statistics for biologists contains articles on many of the points above.

Software and code

Policy information about availability of computer code

Data collection	GraphPad Prism version 7, Adobe Photoshop version CS5, Microsoft Powerpoint version 2010.
Data analysis	GraphPad Prism version 7.

For manuscripts utilizing custom algorithms or software that are central to the research but not yet described in published literature, software must be made available to editors and reviewers. We strongly encourage code deposition in a community repository (e.g. GitHub). See the Nature Research guidelines for submitting code & software for further information.

Data

Policy information about availability of data

All manuscripts must include a data availability statement. This statement should provide the following information, where applicable:

- Accession codes, unique identifiers, or web links for publicly available datasets
- A list of figures that have associated raw data
- A description of any restrictions on data availability

The data that support the findings of this study are available from the corresponding authors upon reasonable request. Source data are provided with this paper.

Field-specific reporting

Life sciences study design

All studies must disclose on these points even when the disclosure is negative.

Sample size	Sample size of mouse experiment was selected based upon mean and standard deviations observed in our previous vaccine studies in mouse models (Liu X et al., NPJ vaccines, 2018). Sample size of rhesus macaque experiment was selected based on our previous study (Feng Y et al., Emerging Microbes & Infections, 2018) and also the limitation of animal biosafety level 4 laboratory. The immunization study includes 4 macaques in each group, which is sufficient for the comparison of different immunization route. The challenge study includes a total of 6 non-vaccinated macaques and 9 vaccinated macaques, which enable an assessment of the protective effects of Ad5-S-nb2 in inhibiting SARS-CoV-2 infection. For in vitro experiments, no sample size calculation was performed. All the biochemical assays and immunofluorescence assay were repeated successfully for at least two times.
Data exclusions	No data was excluded.
Replication	The number of replicates is described and/or shown for each dataset, and supports the reproducibility of the findings.
Randomization	Age and sex matched animals were randomly allocated into each experimental group. The other experiments did not involve any patient, participant or group of people where randomization should be essential to avoid any kind of bias.
Blinding	For the examination of the tissue sections, one-blinded mannar was adopted. The other experiments involving immunological and virological analysis of the animal samples were performed by technicians, who were blinded to the identity and group allocation of the samples. The biochemical assays and immunofluorescence assay did not involve animal or human samples, thus it should be not essential to avoid any kind of bias.

Reporting for specific materials, systems and methods

We require information from authors about some types of materials, experimental systems and methods used in many studies. Here, indicate whether each material, system or method listed is relevant to your study. If you are not sure if a list item applies to your research, read the appropriate section before selecting a response.

Materials & experimental systems

n/a	Involved in the study	
☐	☒	Antibodies
☐	☒	Eukaryotic cell lines
☒	☐	Palaeontology and archaeology
☐	☒	Animals and other organisms
☒	☐	Human research participants
☒	☐	Clinical data
☒	☐	Dual use research of concern

Methods

n/a	Involved in the study	
☒	☐	ChIP-seq
☒	☐	Flow cytometry
☒	☐	MRI-based neuroimaging

Antibodies

Antibodies used

-ELISA
HRP-labeled Goat Anti-Mouse IgG(H+L) (Beyotime, A0216)
Goat Anti-Mouse IgA alpha chain (HRP) (abcam, ab97235)
Goat Anti-Monkey IgG H&L (HRP) (abcam, ab112767)

-Western blot
HRP-labeled Goat Anti-Human IgG(H+L) (Beyotime, A0201)
SARS-CoV Spike Antibody (Sino Biological, 40150-T62-CoV2)
HRP-conjugated goat anti-rabbit IgG (SeraCare, 52200336)
Anti-β-actin (4abio, 4ab082231)
HRP-conjugated goat anti-mouse IgG (SeraCare, 52200341)

-Immunofluorescence assay:
Alexa Fluor 488 AffiniPure Goat Anti-Rabbit IgG (H+L) (Yeasen, 33106ES60)

-ELISpot
Mouse IFN-γ ELISPOT antibody pair (U-CyTech, CT655)
Monkey IFN-γ ELISPOT antibody pair (U-CyTech, CT610)

Validation

-ELISA
HRP-labeled Goat Anti-Mouse IgG(H+L) (Beyotime, A0216): Ref to publication, PMID 29921287;
Goat Anti-Mouse IgA alpha chain (HRP) (abcam, ab97235): Ref to publication, PMID 30867416;
Goat Anti-Monkey IgG H&L (HRP) (abcam, ab112767): Ref to publication, PMID 32318665;

-Western blot
HRP-labeled Goat Anti-Human IgG(H+L) (Beyotime, A0201): Ref to publication, PMID 31853284;
SARS-CoV Spike Antibody (Sino Biological, 40150-T62-CoV2): Ref to publication, Ou X et al., Nat Communic 2020;
HRP-conjugated goat anti-rabbit IgG (SeraCare, 52200336): Ref to publication, PMID 32368013;
Anti-β-actin (4abio, 4ab082231): We validated this polyclonal antibody by WB assays using different cell lines including A549, Hela, HepG2, MDCK etc. In these assays, we used purified human beta actin protein (MyBiosourse, Cat: MBS634566) and the culture supernatants of these cell lines as the positive and negative control, respectively. We also used another validated anti-β-actin antibody (Abcam, Cat. ab8227) as a positive control.
HRP-conjugated goat anti-mouse IgG (SeraCare, 52200341): Ref to publication, PMID 31007733;

-Immunofluorescence assay:
Alexa Fluor 488 AffiniPure Goat Anti-Rabbit IgG (H+L) (Yeasen, 33106ES60): Ref to publication, PMID 29620240;

-ELISpot
Mouse IFN-γ ELISPOT antibody pair (U-CyTech, CT655): Ref to publication, PMID 25252962;
Monkey IFN-γ ELISPOT antibody pair (U-CyTech, CT610): Ref to publication, PMID 31736945;

Eukaryotic cell lines

Policy information about cell lines

Cell line source(s)	Vero (ATCC® CCL-81), HEK293 cell (ATCC® CRL-1573), 293T cell (ATCC® CRL-3216), A549 cell (ATCC® CCL-185), Vero E6 cells (ATCC® CRL-1586)
Authentication	All the cell lines were purchased from American Type Cell Culture (www.atcc.org). These cell lines were authenticated by ATCC, but were not further confirmed during in-house passaging.
Mycoplasma contamination	The cell lines were tested by ATCC and there is no mycoplasma contamination. They were tested monthly once received in-house, and all were tested negative for mycoplasma.
Commonly misidentified lines (See ICLAC register)	No commonly misidentified cell lines were used in this study.

Animals and other organisms

Policy information about studies involving animals; ARRIVE guidelines recommended for reporting animal research

Laboratory animals	Female BALB/c mice at 6–8 weeks of age were purchased from Beijing Vital River Laboratory Animal Technology Co. Ltd. Rhesus macaques at 6-14 years old were purchased from Guangdong Landau Biotechnology Co. Ltd.
Wild animals	The study did not involve wild animals.
Field-collected samples	The study did not involve field-collected samples
Ethics oversight	The vaccination experiments were approved by the Institutional Animal Care and Use Committee (No. 2020025 and 2020009 for mice and macaques, respectively) of Guangzhou Institutes of Biomedicine and Health, Chinese Academy of Sciences. The challenge experiment in rhesus macaques was approved by the IACUC of Wuhan Institute of Virology, Chinese Academy of Sciences(No. WIVA21202002).

Note that full information on the approval of the study protocol must also be provided in the manuscript.
